# Supplementary material for: DiSPA: differential substructure-pathway attention for drug response prediction
Source: Bioinformatics. 2026 Jul 7;42(Suppl 1):btag272. doi: 10.1093/bioinformatics/btag272 (PMC13340222; doi:10.1093/bioinformatics/btag272)
Supplement: btag272_Supplementary_Data [file btag272_supplementary_data.zip › Lim.133.sup.1.pdf]

Supplementary Materials for

DiSPA: Differential Substructure-Pathway Attention for Drug Response Prediction

Yewon Han[1], Sunghyun Kim[2], Eunyi Jeong[2], Sungkyung Lee[3], Seokwoo Yun[4], Sangsoo Lim[3,*]

[1] Division of AI Software Convergence, Dongguk University, 04620, 30, Pildong-ro 1-gil, Jung-gu, Seoul, South Korea

[2] Division of AI Convergence, Dongguk University, 04620, 30, Pildong-ro 1-gil, Jung-gu, Seoul, South Korea

[3] Department of Computer Science and Artificial Intelligence, Dongguk University, 04620, 30, Pildong-ro 1-gil, Jung-gu, Seoul, South Korea

[4] AI Research Team, Ar-ge Inc., 08757, 408-1, Bongcheon-ro, Gwanak-gu, Seoul, South Korea

[*]Corresponding author Email: sslim@dgu.ac.kr

Keywords: drug response prediction, pharmacogenomics, differential cross-attention, biological pathways, chemical substructures, representation learning

Supplementary Approaches

Approach S1. Data availability.

Approach S2. Data preprocessing.

Approach S3. Comparison with other drug response prediction methods.

Supplementary Figures

Figure S1. Scaffold similarity between test drugs and the training set across splits.

Figure S2. Gene expression similarity across data split settings.

Figure S3. Fold-wise RMSE distributions and scaffold similarity analysis in the drug-blind setting.

Figure S4. Quantitative comparison of attention concentration between vanilla and differential cross-attention.

Figure S5. Cumulative attention mass comparison between vanilla and differential cross-attention.

Figure S6. Comparison of pathway prioritization under vanilla and differential cross-attention.

Figure S7. Additional case studies of substructure-pathway interactions captured by DiSPA.

Figure S8. Top 5 domain-specific drug response analyses in the IDC spatial transcriptomics dataset.

Figure S9. Manual annotation results and spot distribution in the IDC dataset.

Figure S10. Cell type-wise UMAP in the CRC single-cell RNA-seq atlas.

Figure S11. Cell subtype-wise UMAP in the CRC single-cell RNA-seq atlas.

Supplementary Tables

Table S1. Pathway-gene mapping for KEGG category 6 pathways.

Table S2. Category-wise performance across KEGG pathway groups.

Table S3. Comparison of full KEGG pathways and category 6 pathway subset.

Table S4. Drugs excluded from BRICS decomposition.

Table S5. Performance comparison under random and drug-blind splits across 5 seeds.

Table S6. Performance on the CTRP dataset (within-dataset evaluation).

Table S7. Cross-dataset generalization.

Table S8. Drug list stratified by substructure-attention alignment.

Table S9. Literature support for tumor-selective compounds identified in the IDC dataset.

Table S10. Comparison of interaction modules.

Table S11. Controlled ablation of vanilla and differential cross-attention across multiple datasets.

Table S12. Component ablation within DiSPA.

Supplementary Methods

Method S1. Scaffold-level similarity analysis.

Approach S1. Data availability.

The source code is publicly available at https://github.com/sslim-aidrug/DiSPA. Gene expression profiles and drug response data (IC_{50}) were obtained from the Genomics of Drug Sensitivity in Cancer (GDSC) database (https://www.cancerrxgene.org/). We used the curated and preprocessed GDSC datasets provided by Shen et al. [1], which are publicly available via Zenodo at https://zenodo.org/records/7060305. Additional drug response information for the CTRPv2 dataset (https://portals.broadinstitute.org/ctrp.v2.1/) was accessed via the PharmacoGx R package. For cross-dataset evaluation, we additionally used an integrated dataset derived from CCLE and PRISM [2], [3] as provided by Chen et al. [4].

Human breast cancer spatial transcriptomics data generated using the 10x Genomics Visium platform were used, including the HER2-amplified invasive ductal carcinoma (IDC) dataset, which is publicly available at https://www.10xgenomics.com/datasets/human-breast-cancer-IDC.

Single-cell RNA-sequencing data were obtained from the Gene Expression Omnibus (GEO) under accession number GSE132465 (https://www.ncbi.nlm.nih.gov/geo/query/acc.cgi?acc=GSE132465).

Approach S2. Data preprocessing.

Drug response data

Genomics of Drug Sensitivity in Cancer (GDSC) (Nucleic Acids Research, 2013*)*

The Genomics of Drug Sensitivity in Cancer (GDSC) dataset [5] provides drug sensitivity measurements ($\ln(IC_{50})$) and gene expression profiles across human cancer cell lines. Drug SMILES were decomposed into BRICS substructures, excluding drugs with unsuccessful decomposition or metal elements, resulting in 270 retained drugs out of 282 total drugs. Transcriptomic features were defined using 1,692 genes from KEGG pathway category 6 and standardized by gene-wise z-score normalization across cell lines. A total of 224,078 drug–cell line response samples with complete information were used for model training and evaluation.

Cancer Cell Line Encyclopedia (CCLE) (Nature, 2012*)*

To integrate CTRPv2 drug response data with transcriptomic profiles, CTRPv2 cell lines were mapped to corresponding entries in the Cancer Cell Line Encyclopedia (CCLE) [2], with 818 of 887 cell lines successfully retained. Transcriptomic features were aligned to 1,692 genes from KEGG pathway category 6, of which expression values were available for 1,600 genes, while the remaining genes were zero-padded and excluded by masking. Gene expression values were standardized using gene-wise z-score normalization across cell lines.

Cancer Therapeutics Response Portal (CTRPv2) (Cancer Discovery, 2015*)*

The Cancer Therapeutics Response Portal v2 (CTRPv2) [6] was used as an independent external dataset for model evaluation. Drug SMILES were decomposed into BRICS substructures, excluding drugs with unsuccessful decomposition or ambiguous isomeric annotations, resulting in 515 retained drugs. Drug response values were transformed using $\ln(IC_{50})$, and extreme values were removed using the interquartile range (IQR) rule. After filtering for drugs and cell lines with available CCLE expression profiles, 205,749 drug–cell line response samples were retained for analysis.

Spatial transcriptomics data

HER2-Amplified Invasive Ductal Carcinoma (IDC) (Nature Communications, 2023*)*

The human breast cancer spatial transcriptomics dataset of HER2-amplified invasive ductal carcinoma (IDC) [7] consists of 3,798 spatial spots (55 μm diameter) profiling 36,591 genes. The four-category morphological annotations provided in the original dataset were used to define spatial tissue domains, as shown in Supplementary Figure S9.

Spot-level gene expression profiles were smoothed in an annotation-aware manner to account for sparse single-cell expression patterns, using the eight immediate neighbors defined by the grid-based Visium layout. The resulting expression profiles were normalized by library size, log-transformed using $\log(1+x)$, and standardized by gene-wise z-score normalization with statistics estimated from the GDSC training set. Drug response predictions were then generated for each spatial spot across all drugs used during GDSC model training.

Single-cell RNA sequencing data

Gene Expression Omnibus *(Nature Genetics,* 2020*)*

The single-cell RNA-sequencing dataset was obtained from the Gene Expression Omnibus (GEO) under accession number GSE132465 [8]. This dataset comprises 63,689 cells from 23 Korean colorectal cancer patients, including samples from 23 primary tumors and 10 matched normal mucosa tissues. Cells are annotated into 6 major cell types (T cells, epithelial cells, B cells, myeloid cells, stromal cells, and mast cells) and 36 cell subtypes.

As gene expression values were already provided in log-transformed form, preprocessing consisted of gene filtering to the target gene set used in the GDSC setting, followed by gene-wise z-score normalization using the mean and standard deviation estimated from the GDSC training set. Drug response predictions were generated for each single cell across all drugs used during GDSC model training.

Approach S3. Comparison with other drug response prediction methods.

We compared DiSPA with 4 recently developed drug response prediction models: DEERS, DeepTTA, DRPreter, and DIPK, which represent diverse modeling strategies and data integration paradigms in the field.

- DEERS (*Scientific Reports*, 2021): DEERS is a deep neural network–based recommender system specifically designed for predicting the sensitivity of kinase inhibitors. The model applies autoencoders to reduce the dimensionality of both cell line features and drug features, followed by a feed-forward neural network for response prediction. A key strength of DEERS is its emphasis on interpretability, achieved by associating latent representations with biological processes.
- DeepTTA (*Bioinformatics*, 2022): DeepTTA is a transformer-based deep learning model for cancer drug response prediction. It is notable as one of the first approaches to apply transformer architectures to learn drug representations directly from chemical substructures. These drug embeddings are then integrated with transcriptomic profiles using a multilayer neural network to predict drug sensitivity.
- DRPreter (*International Journal of Molecular Sciences*, 2022): DRPreter is an interpretable anticancer drug response prediction framework that combines knowledge-guided graph neural networks with transformer architectures. Beyond accurate prediction, DRPreter is designed to elucidate underlying biological mechanisms by identifying and highlighting relevant signaling pathways that contribute to drug sensitivity.
- DIPK (*Briefings in Bioinformatics*, 2024): DIPK is a comprehensive deep learning framework that integrates gene interaction networks, gene expression profiles, and molecular topology information. It employs self-supervised learning strategies and multi-head attention mechanisms to improve prediction accuracy and robustness. DIPK is particularly well suited for handling heterogeneous data types, including single-cell RNA sequencing data.

For all baseline methods, hyperparameters were tuned separately for each benchmark to ensure fair comparison.

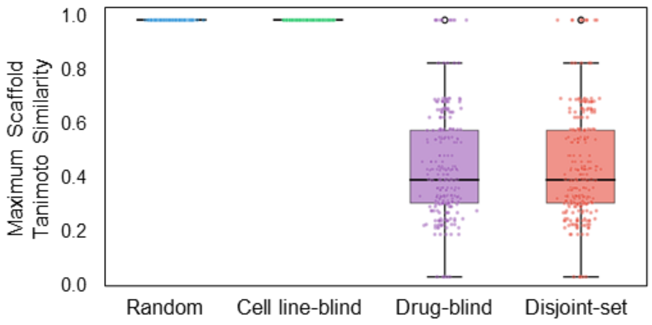

Figure S1. Scaffold similarity between test drugs and the training set across splits.

Across data splits, scaffold similarity for each test drug was defined as the maximum Tanimoto similarity between its scaffold fingerprint and those of training drugs. See Method S1 for details of scaffold similarity calculation.

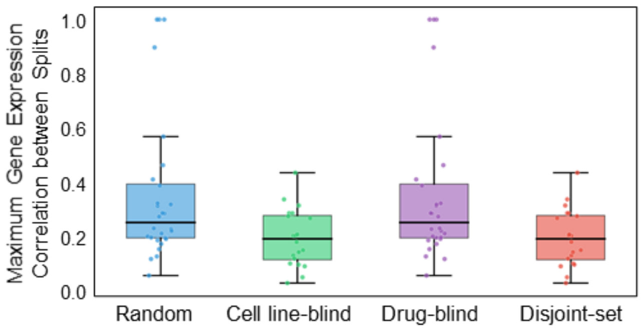

Figure S2. Gene expression similarity across data split settings.

Maximum gene expression correlation between training and test samples under random, drug-blind, cell line-blind, and disjoint-set splits.

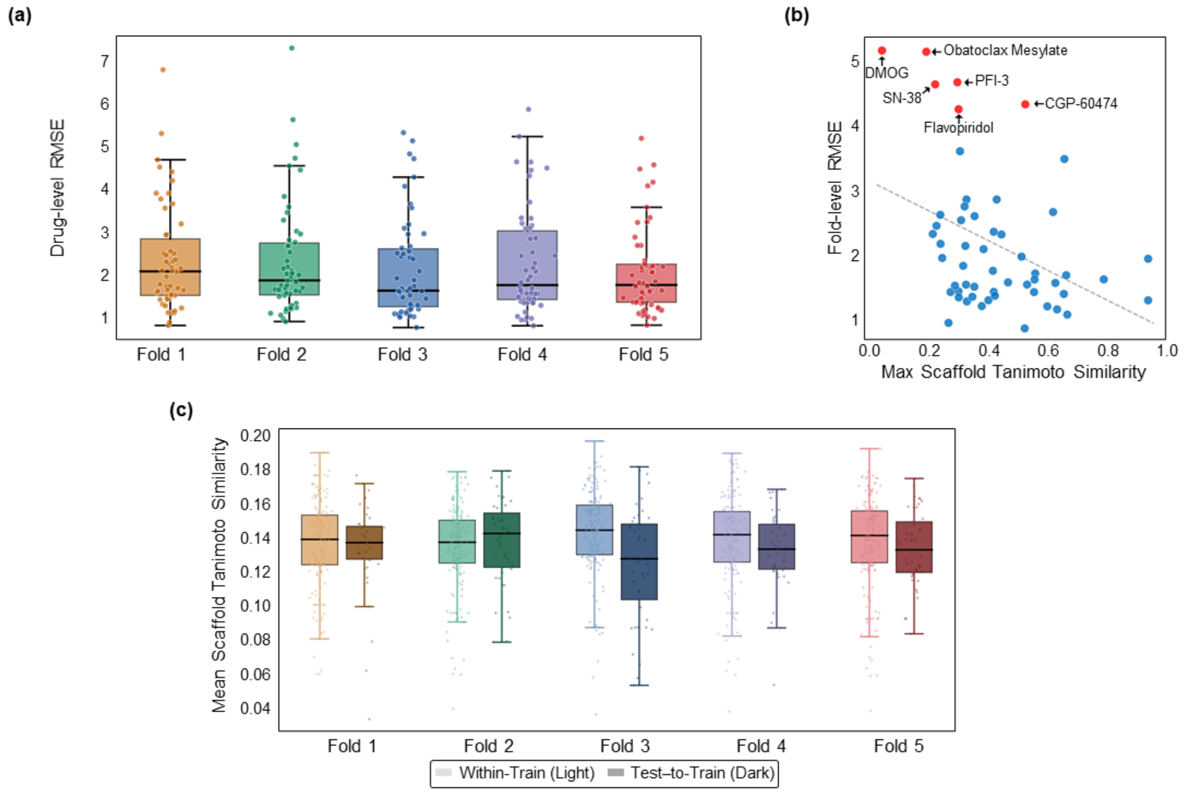

Figure S3. Fold-wise RMSE distributions and scaffold similarity analysis in the drug-blind setting.

(a) Distribution of drug-level RMSE across 5 folds. (b) Relationship between drug-level RMSE and maximum scaffold Tanimoto similarity to training drugs. (c) Comparison of within-train and test-to-train scaffold similarity across folds.

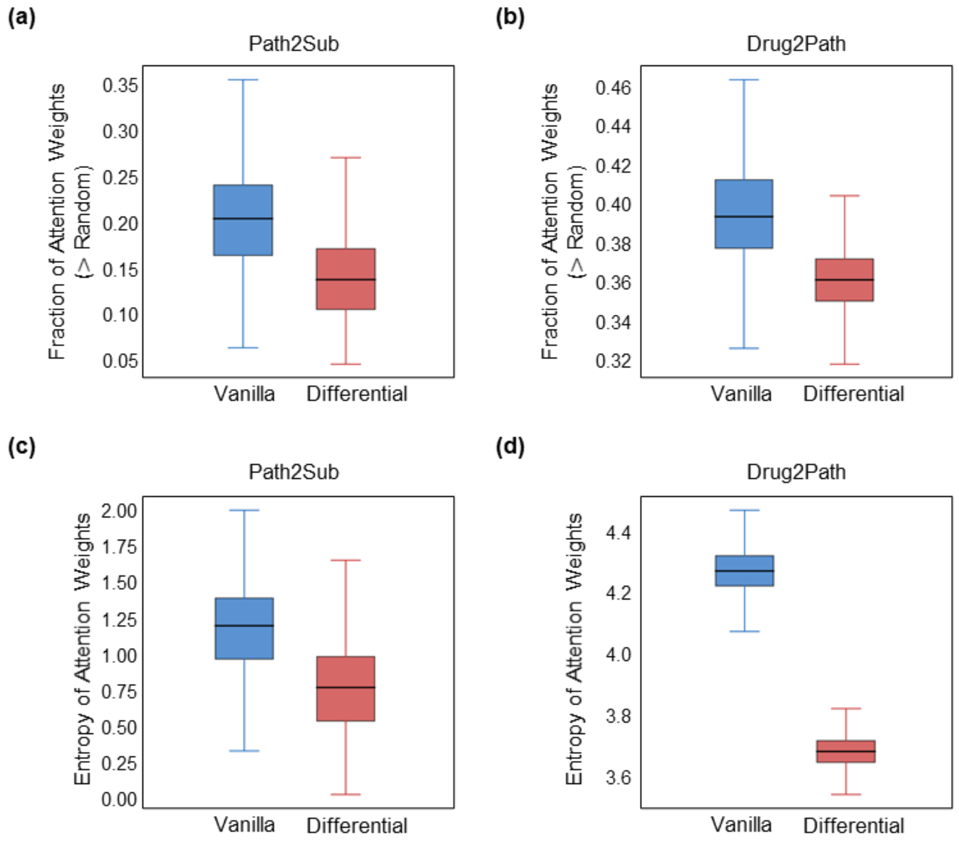

Figure S4. Quantitative comparison of attention concentration between vanilla and differential cross-attention.

Panels (a,b) present head-averaged sparsity, defined as the fraction of attention values above the random attention level, and panels (c,d) present head-averaged Shannon entropy. Lower values in both metrics indicate that attention is distributed over fewer entries and is therefore more concentrated.

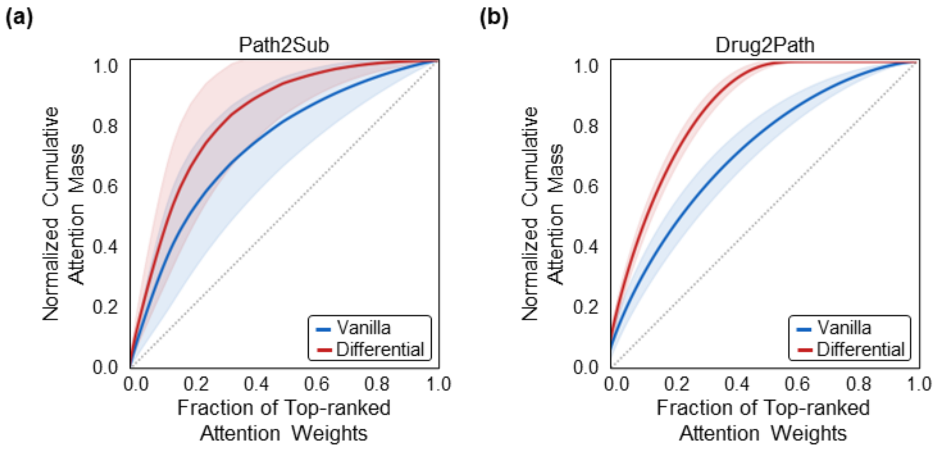

Figure S5. Cumulative attention mass comparison between vanilla and differential cross-attention.

Cumulative fraction of attention weights (sorted in descending order) for pathway–substructure attention maps in Path2Sub (a) and Drug2Path (b).

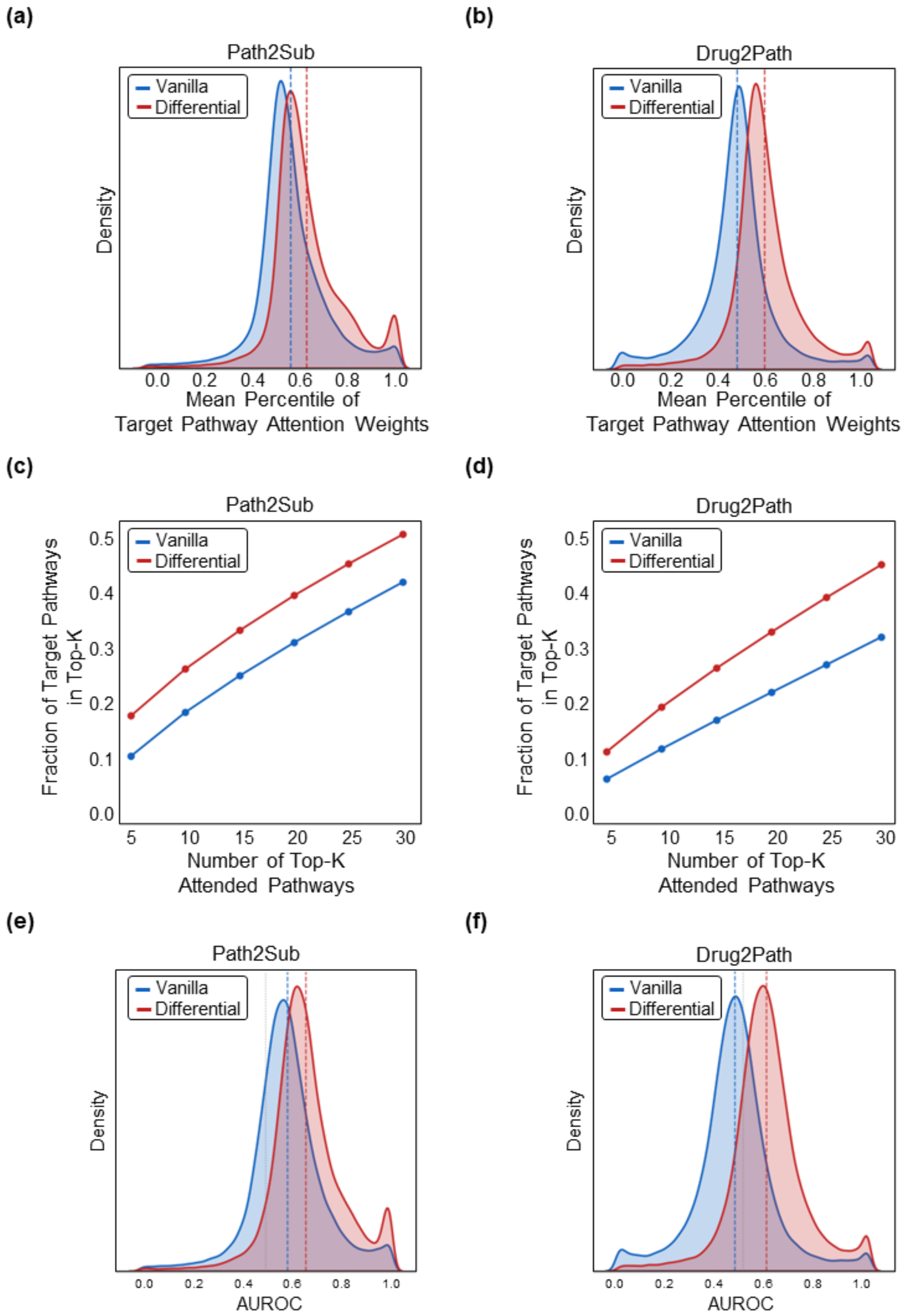

Figure S6. Comparison of pathway prioritization under vanilla and differential cross-attention.

Distributions of mean target pathway percentile (a,b), Top-K inclusion (c,d), and AUROC (e,f) for target pathways derived from Path2Sub and Drug2Path modules. Differential cross-attention consistently shows improved ranking of target pathways compared to vanilla cross-attention.

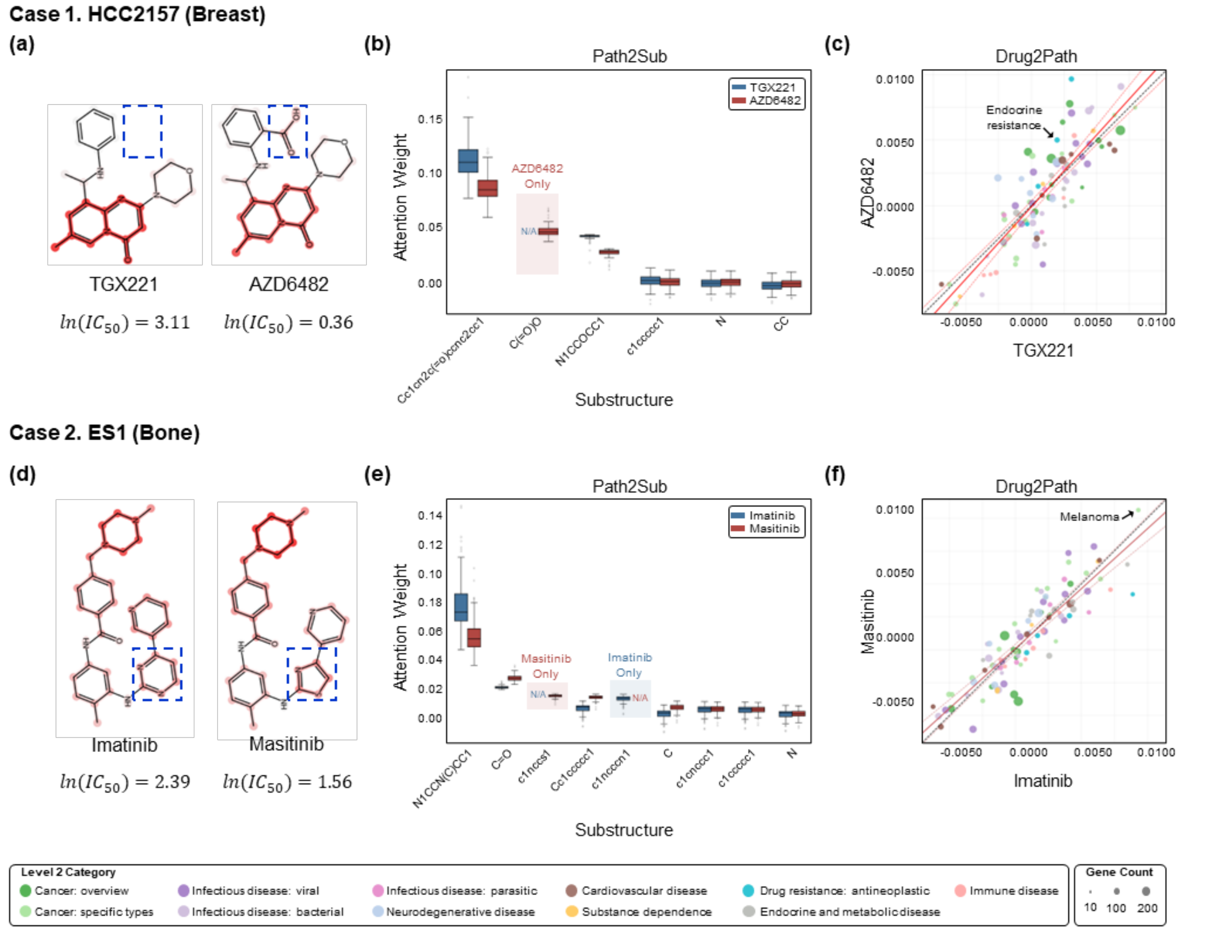

Figure S7. Additional case studies of substructure-pathway interactions captured by DiSPA.

(a,d) Chemical structures of paired drugs evaluated in the same cell line, with highlighted substructures (blue dashed boxes) exhibiting differential attention patterns. Predicted drug sensitivities are reported as ($\ln(IC_{50})$). (b,e) Substructure-level attention weights aggregated over biological pathways (Path2Sub), showing differential emphasis on specific substructures between drug pairs. Substructures exhibiting drug-specific attention patterns are annotated. (c,f) Drug-to-pathway (Drug2Path) attention comparisons across pathways, illustrating pathway-level response differences between paired drugs. Each point represents a biological pathway, colored by level 2 disease category and scaled by the number of associated genes. Top: Case 1 (HCC2157, breast cancer) comparing TGX221 and AZD6482. Bottom: Case 2 (ES1, bone cancer) comparing imatinib and masitinib. These examples demonstrate how DiSPA captures both shared and drug-specific structure-pathway interactions that correspond to distinct sensitivity profiles across biological contexts.

Healthy	LDN-193189	FTY-720	CX-5461	Gemcltablne	Flavopiridol
CI ($\ln(IC_{50})$)	[-0.0279, -0.0232]	[-0.0221, -0.0171]	[-0.0882, -0.0665]	[-0.0911, -0.0760]	[-0.0499, -0.0413]
p-value	8.56e-126	1.82e-99	1.17e-82	1.16e-77	3.16e-77
Moran's I	0.8150	0.7593	0.7135	0.7651	0.7748

Spatial Visualization

$\ln(IC_{50})$ Boxplot

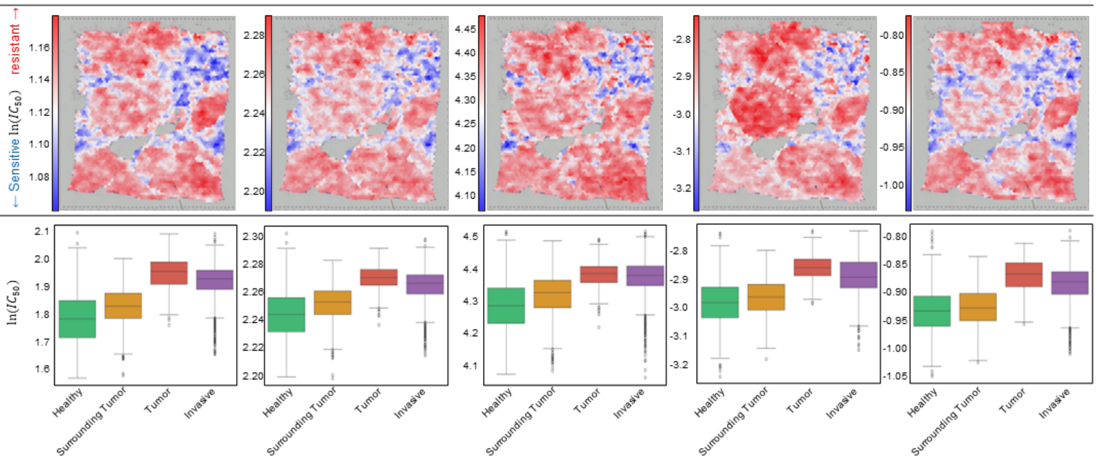

Surrounding Tumor	CI-1040	NU7441	Nilotinib	Temslrollmus	LDN-193189
CI ($\ln(IC_{50})$)	[-0.0900, -0.0767]	[-0.0315, -0.0269]	[-0.0261, -0.0217]	[-0.0712, -0.0587]	[-0.0279, -0.0232]
p-value	3.15e-136	1.15e-132	6.31e-129	4.58e-125	6.96e-125
Moran's I	0.8173	0.7681	0.7258	0.7419	0.8150

Spatial Visualization

$\ln(IC_{50})$ Boxplot

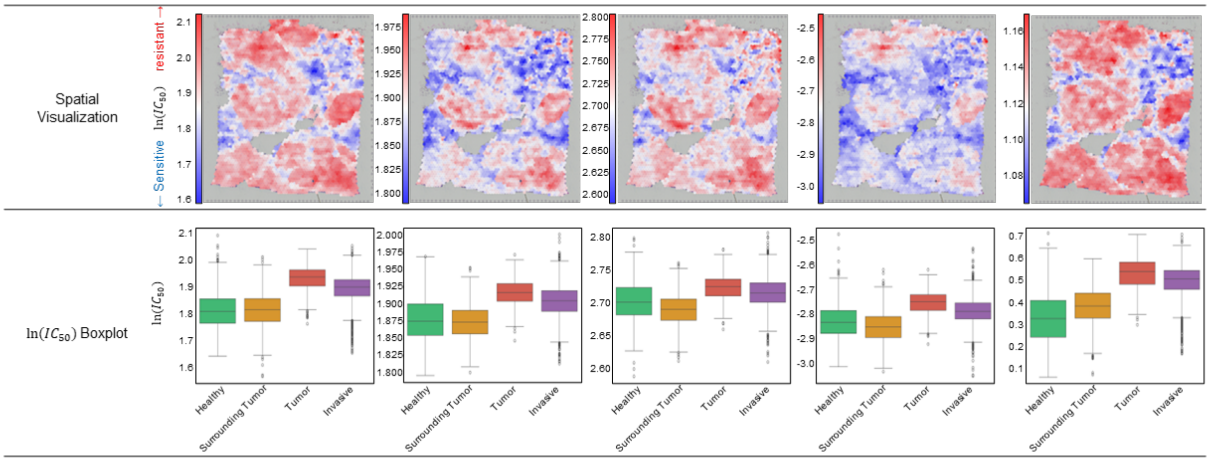

See next page

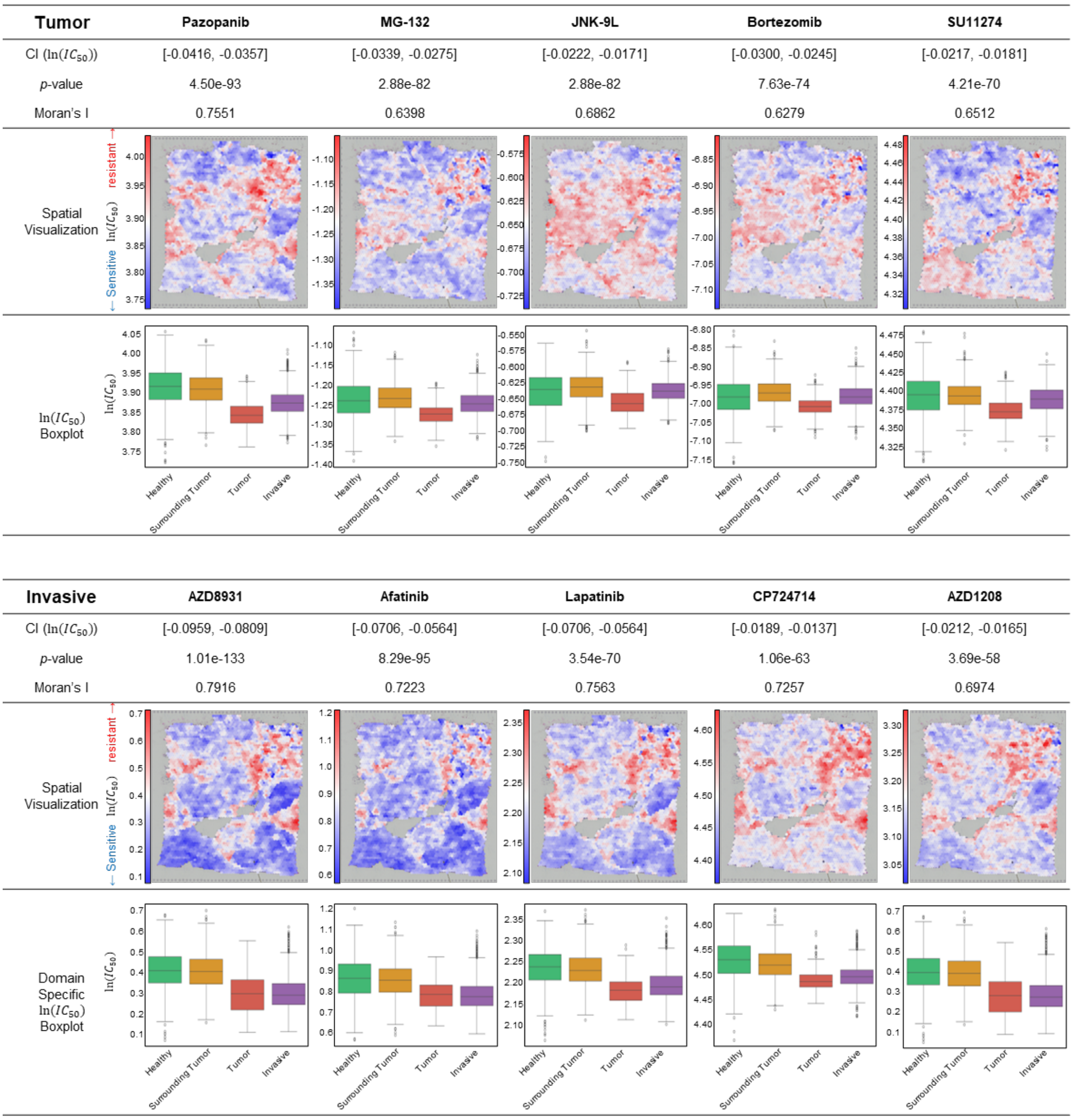

Figure S8. Top 5 domain-specific drug response analyses in the IDC spatial transcriptomics dataset.

Supplementary Figure S8 presents comprehensive domain-specific analyses of the top 5 selective drugs across all spatial compartments in the IDC dataset, showing statistically significant domain-wise differences in predicted $\ln(IC_{50})$ distributions with coherent, spatially localized sensitivity patterns supported by confidence intervals and elevated Moran's I statistics.

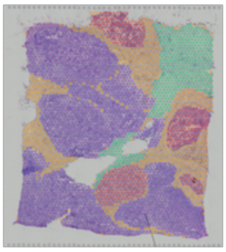

Spatial Domain Label	Number of Spots
Healthy	485
Surrounding Tumor	823
Tumor	463
Invasive	2,027
**Total**	3,798

Figure S9. Manual annotation results and spot distribution in the IDC dataset.

The table summarizes the number of spatial spots assigned to each annotation category.

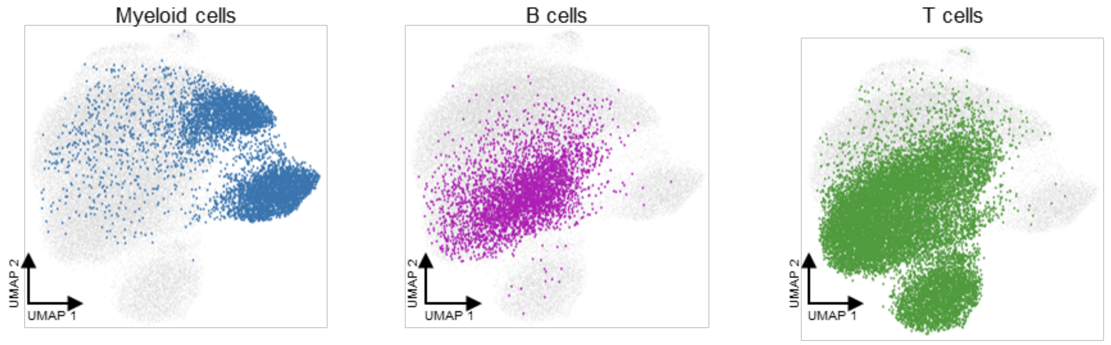

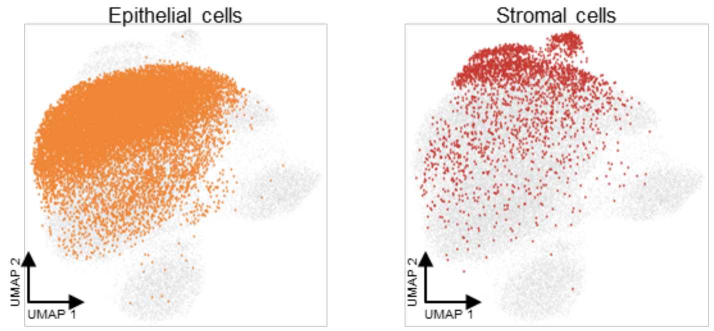

Cell Type	Number of Cells
Myeloid cells	6,400
B cells	3,938
T cells	16,739
Epithelial cells	17,469
Stromal cells	2,736
**Total**	47,282

Figure S10. Cell type-wise UMAP in the CRC single-cell RNA-seq atlas.

Cell embeddings were constructed from DiSPA-predicted drug response ($\ln(IC_{50})$) profiles using PCA followed by UMAP in the colorectal cancer single-cell RNA-seq atlas. Each panel displays the same shared UMAP embedding, with cells of a specific annotated cell type highlighted to visualize their distribution within the embedding space. The accompanying table summarizes the number of cells for each cell type.

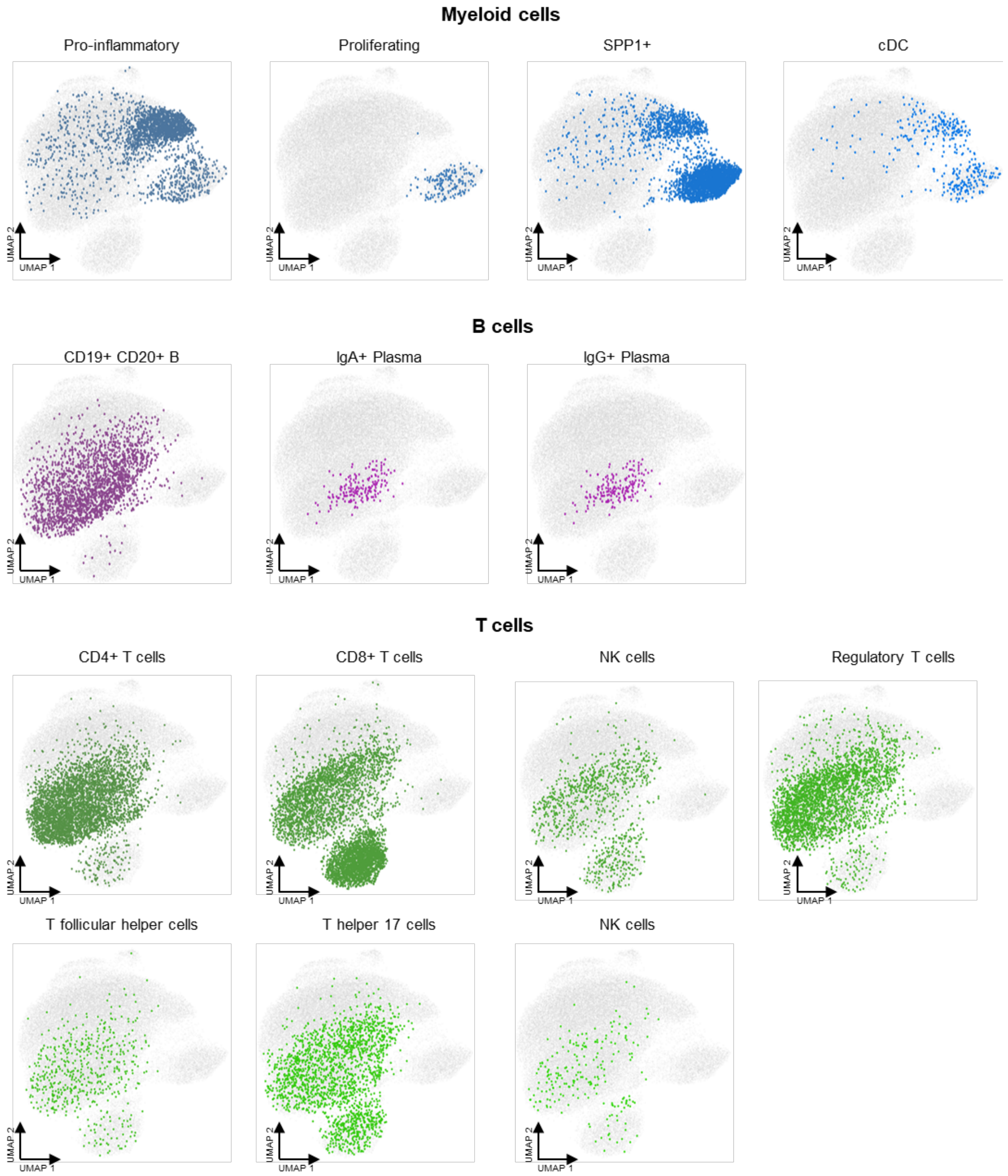

See next page

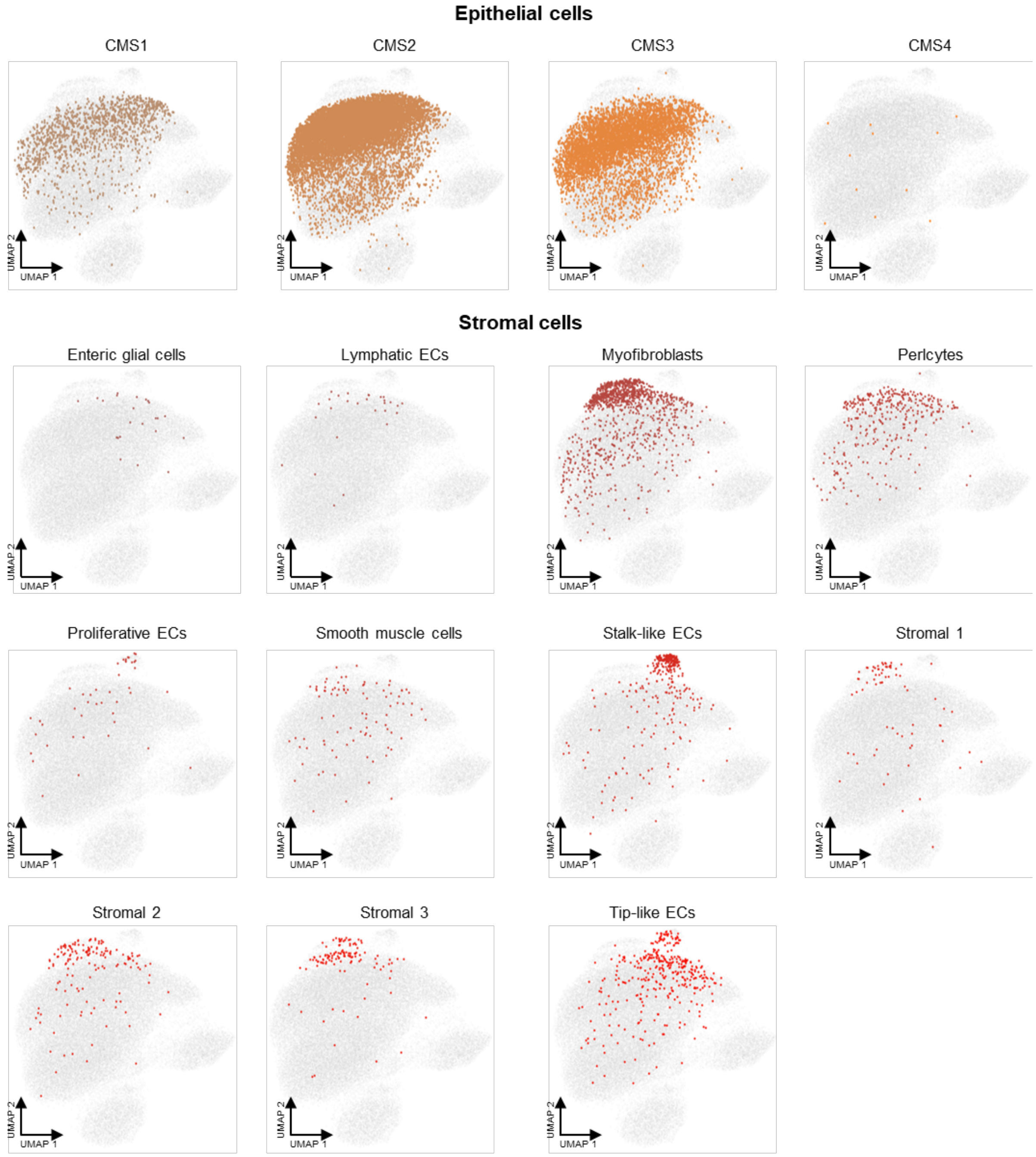

Figure S11. Cell subtype-wise UMAP in the CRC single-cell RNA-seq atlas.

Cell embeddings were constructed from DiSPA-predicted drug response ($\ln(IC_{50})$) profiles using PCA followed by UMAP in the colorectal cancer single-cell RNA-seq atlas. Each panel displays the same shared UMAP embedding, with cells of a specific annotated cell subtype highlighted to visualize their distribution within the embedding space, enabling visualization of intra-cell-type heterogeneity.

Table S1. Pathway-gene mapping for KEGG category 6 pathways.

Pathway IDs, KEGG level 3 pathways, gene counts, and mapped genes for the 94 pathways used in DiSPA.

Table S2. Category-wise performance across KEGG pathway groups.

DiSPA performance (RMSE, PCC, SCC) using pathway inputs constructed from each KEGG top-level category. Results are reported as mean ± s.d. across runs.

**KEGG pathway category**	**RMSE (↓)**	**PCC (↑)**	**SCC (↑)**
1 Metabolism	0.9264 ± 0.0064	0.9373 ± 0.0008	0.9205 ± 0.0009
2 Genetic Information Processing	0.9222 ± 0.0093	0.9377 ± 0.0013	0.9210 ± 0.0015
3 Environmental Information Processing	0.9084 ± 0.0060	0.9396 ± 0.0007	0.9229 ± 0.0010
4 Cellular Processes	0.9125 ± 0.0045	0.9390 ± 0.0005	0.9226 ± 0.0007
5 Organismal Systems	0.9148 ± 0.0066	0.9387 ± 0.0009	0.9224 ± 0.0009
**6 Human Diseases**	**0.9059 ± 0.0023**	**0.9399 ± 0.0004**	**0.9236 ± 0.0003**

Table S3. Comparison of full KEGG pathways and category 6 pathway subset.

Performance (RMSE, PCC, SCC) and computational cost (time per epoch, peak GPU memory) of DiSPA using all KEGG pathways (n = 314) versus category 6 pathways (n = 94). Results are reported as mean ± s.d. across runs.

**KEGG pathway set (# of pathways)**	**Time per epoch**	**Peak GPU memory**	**RMSE (↓)**	**PCC (↑)**	**SCC (↑)**
Full category (314)	26.8s	11.21GB	0.9264 ± 0.0064	0.9373 ± 0.0008	0.9205 ± 0.0009
**Category 6 (94)**	**11.7s**	**3.66GB**	**0.9059 ± 0.0023**	**0.9399 ± 0.0004**	**0.9236 ± 0.0003**

Table S4. Drugs excluded from BRICS decomposition.

This table summarizes the drugs excluded from the BRICS decomposition step.

Table S5. Performance comparison under random and drug-blind splits across 5 seeds.

Mean ± s.d. of RMSE, PCC, and SCC are reported across seeds as performance, together with fold-level variability as stability, calculated using the geometric mean of standard deviations. Lower RMSE indicates better accuracy, and higher PCC/SCC indicate better concordance.

**Random**	**RMSE**		**PCC**		**SCC**	
	**Performance (↓)**	**Stability (↓)**	**Performance (↑)**	**Stability (↓)**	**Performance (↑)**	**Stability (↓)**
DEERS	1.6435 ± 0.0125	0.0176	0.8422 ± 0.0015	0.0040	0.8955 ± 0.0007	0.0018
DRPreter	<u>0.9474 ± 0.0077</u>	0.0067	<u>0.9337 ± 0.0011</u>	0.0010	<u>0.9158 ± 0.0008</u>	0.0013
DeepTTA	0.9605 ± 0.0039	<u>0.0043</u>	0.9320 ± 0.0007	<u>0.0006</u>	0.9139 ± 0.0005	<u>0.0007</u>
DIPK	0.9636 ± 0.0024	0.0058	0.9316 ± 0.0006	0.0007	0.9137 ± 0.0008	0.0009
**DiSPA (proposed)**	**0.9025 ± 0.0049**	**0.0029**	**0.9403 ± 0.0007**	**0.0004**	**0.9227 ± 0.0007**	**0.0006**

**Drug-blind**	**RMSE**		**PCC**		**SCC**	
	**Performance (↓)**	**Stability (↓)**	**Performance (↑)**	**Stability (↓)**	**Performance (↑)**	**Stability (↓)**
DEERS	2.3476 ± 0.1923	**0.0264**	0.3873 ± 0.0871	**0.0265**	<u>0.3673 ± 0.0811</u>	**0.0266**
DRPreter	<u>2.3446 ± 0.2051</u>	0.1060	<u>0.4174 ± 0.1151</u>	0.0609	0.3253 ± 0.0888	0.0576
DeepTTA	2.4801 ± 0.1973	0.0993	0.3357 ± 0.0606	0.0698	0.2685 ± 0.0658	0.0638
DIPK	2.4520 ± 0.2357	0.0861	0.4061 ± 0.0792	0.0465	0.3139 ± 0.0580	<u>0.0331</u>
**DiSPA (proposed)**	**2.3384 ± 0.1860**	<u>0.0746</u>	**0.4451 ± 0.0625**	<u>0.0416</u>	**0.3679 ± 0.0386**	0.0355

Table S6. Performance on the CTRP dataset (within-dataset evaluation).

DiSPA and baseline models trained and evaluated on CTRP. Results are reported as mean ± s.d.; DiSPA achieves the best overall performance.

**Model**	**RMSE (↓)**	**PCC (↑)**	**SCC (↑)**
DEERS	1.4269 ± 0.0290	0.7915 ± 0.0053	0.8308 ± 0.0060
DRPreter	1.0607 ± 0.0225	0.8855 ± 0.0054	0.8901 ± 0.0055
DeepTTA	1.0793 ± 0.0019	0.8824 ± 0.0007	0.8873 ± 0.0006
DIPK	1.0525 ± 0.0040	0.8872 ± 0.0008	0.8921 ± 0.0014
**DiSPA (proposed)**	**1.0506 ± 0.0034**	**0.8881 ± 0.0008**	**0.8941 ± 0.0008**

Table S7. Cross-dataset generalization.

Number of drugs with significant separation between predicted sensitive and resistant samples ($p < 0.01$ and $p < 0.05$), using fold-averaged predictions, on an integrated dataset from CCLE and PRISM (476 cell lines; 78 drugs).

**Significant drugs**	**p < 0.01**	**p < 0.05**
DEERS	30.6 ± 2.2	41.0 ± 1.7
DRPreter	40.6 ± 0.5	46.2 ± 1.7
DeepTTA	27.0 ± 0.6	34.6 ± 2.3
DIPK	38.0 ± 1.1	44.6 ± 1.4
**DiSPA (proposed)**	**41.4 ± 1.0**	**47.6 ± 1.4**

Table S8. Drug list stratified by substructure-attention alignment.

The table provides a detailed list of drugs stratified according to their substructure-attention alignment, which quantifies the degree to which learned substructure-level attention patterns correspond to intrinsic chemical substructure similarity. This table serves as a reference for all analyses involving high and low alignment drug groups.

Table S9. Literature support for tumor-selective compounds identified in the IDC dataset.

Summary of prior experimental or clinical evidence linking predicted tumor-selective compounds to breast cancer.

Drug	Breast cancer relevance	Summary
Pazopanib	Recurrent or metastatic invasive breast cancer	Pazopanib monotherapy demonstrated disease stabilization in a substantial proportion of patients with recurrent or metastatic breast cancer, with measurable tumor shrinkage observed in a subset, supporting its cytostatic anti-tumor activity in advanced disease [9].
MG-132	Breast cancer (chromatin reprogramming)	In ER-positive breast cancer cells, proteasome inhibition using MG-132 led to widespread reprogramming of chromatin accessibility and transcription initiation at regulatory regions, highlighting a mechanistic link between proteasome activity and oncogenic transcriptional regulation [10].
JNK-9L	HER2-positive breast cancer	Disruption of JNK signaling accelerated tumor progression in HER2-driven breast cancer models, indicating that JNK activity plays a tumor-suppressive role rather than serving as a straightforward therapeutic inhibition target [11].
Bortezomib	ER-positive metastatic breast cancer	Combination of the proteasome inhibitor bortezomib with fulvestrant showed improved progression-related outcomes in a subset of patients with endocrine-resistant ER-positive metastatic breast cancer, suggesting a role for proteasome inhibition in overcoming acquired endocrine resistance [12].
SU11274	Triple-negative breast cancer	In triple-negative breast cancer cells, SU11274 contributed to reduced cell survival and induced G2 arrest, particularly in combination with EGFR inhibition, supporting relevance in aggressive breast cancer subtypes [13].

Table S10. Comparison of interaction modules.

The table compares interaction modules under the same encoders, inputs, and training protocol.

**Interaction module setting**	**RMSE (↓)**	**PCC (↑)**	**SCC (↑)**
Late fusion	1.1653 ± 0.1333	0.8985 ± 0.0210	0.8768 ± 0.0238
Simple gated fusion	0.9769 ± 0.1402	0.9294 ± 0.0208	0.9118 ± 0.0238
**DiSPA (Differential cross-attention)**	**0.9059 ± 0.0023**	**0.9399 ± 0.0004**	**0.9236 ± 0.0003**

Table S11. Controlled ablation of vanilla and differential cross-attention across multiple datasets.

This table presents a controlled comparison of vanilla and differential cross-attention under identical settings, evaluated on GDSC and CTRP.

**Dataset**	**RMSE (↓)**	**PCC (↑)**	**SCC (↑)**
**GDSC**			
Vanilla cross-attention	0.9102 ± 0.0070	0.9394 ± 0.0009	0.9229 ± 0.0010
DiSPA (Differential cross-attention)	**0.9059 ± 0.0023**	**0.9399 ± 0.0004**	**0.9236 ± 0.0003**
**CTRP**			
Vanilla cross-attention	1.0525 ± 0.0102	0.8880 ± 0.0021	0.8937 ± 0.0021
DiSPA (Differential cross-attention)	**1.0506 ± 0.0034**	**0.8881 ± 0.0008**	**0.8941 ± 0.0008**

Table S12. Component ablation within DiSPA.

The table reports the performance of DiSPA under various ablation settings, evaluating both input-level and module-level components. For input-level ablations, the study is conducted by removing each of the branches used in the final prediction, one at a time. For module-level ablations, the Path2Sub and Drug2Path settings remove the corresponding attention modules and instead use simple embedding-based representations. Performance is evaluated using RMSE, PCC, and SCC.

**Setting**	**RMSE (↓)**	**PCC (↑)**	**SCC (↑)**
Input-level Ablation			
w/o Pathway embedding	1.3683 ± 0.0002	0.8557 ± 0.0000	0.8244 ± 0.0001
w/o Drug, Substructure embedding	2.5754 ± 0.0010	0.2271 ± 0.0016	0.2310 ± 0.0016
w/o Drug embedding	0.9146 ± 0.0034	0.9388 ± 0.0004	0.9225 ± 0.0006
w/o Substructure embedding	0.9625 ± 0.0034	0.9316 ± 0.0004	0.9143 ± 0.0004
Module-level Ablation			
w/o Path2Sub, Drug2Path	0.9743 ± 0.1436	0.9294 ± 0.0224	0.9115 ± 0.0260
w/o Path2Sub	0.9147 ± 0.0029	0.9385 ± 0.0005	0.9219 ± 0.0008
w/o Drug2Path	0.9656 ± 0.0032	0.9317 ± 0.0003	0.9137 ± 0.0005
**DiSPA (proposed)**	**0.9059 ± 0.0023**	**0.9399 ± 0.0004**	**0.9236 ± 0.0003**

Method S1. Scaffold-level similarity analysis.

Within each split, scaffold-level similarity for each test drug was defined as the maximum Tanimoto similarity between its scaffold fingerprint and those of training drugs:

$$Scaffold\ Similarity(d_t) = \max_{d_r \in D_{train}} Tanimoto\left(\varphi_{scaf}(dt), \varphi_{scaf}(d_r)\right)$$

where d_t denotes a test drug and D_{train} denotes the corresponding training set.

Supplementary References

[1] Shen, Bihan, et al. "A systematic assessment of deep learning methods for drug response prediction: from in vitro to clinical applications." *Briefings in Bioinformatics* 24.1 (2023): bbac605.

[2] Barretina, Jordi, et al. "The Cancer Cell Line Encyclopedia enables predictive modelling of anticancer drug sensitivity." *Nature* 483.7391 (2012): 603-607.

[3] Yu, Channing, et al. "High-throughput identification of genotype-specific cancer vulnerabilities in mixtures of barcoded tumor cell lines." *Nature Biotechnology* 34.4 (2016): 419-423.

[4] Chen, Junyi, et al. "Deep transfer learning of cancer drug responses by integrating bulk and single-cell RNA-seq data." *Nature Communications* 13.1 (2022): 6494.

[5] Yang, Wanjuan, et al. "Genomics of Drug Sensitivity in Cancer (GDSC): a resource for therapeutic biomarker discovery in cancer cells." *Nucleic Acids Research* 41.D1 (2012): D955-D961.

[6] Seashore-Ludlow, Brinton, et al. "Harnessing connectivity in a large-scale small-molecule sensitivity dataset." *Cancer Discovery* 5.11 (2015): 1210-1223.

[7] Xun, Zhenzhen, et al. "Reconstruction of the tumor spatial microenvironment along the malignant-boundary-nonmalignant axis." *Nature Communications* 14.1 (2023): 933.

[8] Lee, Hae-Ock, et al. "Lineage-dependent gene expression programs influence the immune landscape of colorectal cancer." *Nature Genetics* 52.6 (2020): 594-603.

[9] Taylor, Sara K., et al. "A phase II study of pazopanib in patients with recurrent or metastatic invasive breast carcinoma: a trial of the Princess Margaret Hospital phase II consortium." *The Oncologist* 15.8 (2010): 810-818.

[10] Kinyamu, H. Karimi, et al. "Proteasome inhibition reprograms chromatin landscape in breast cancer." *Cancer Research Communications* 4.4 (2024): 1082-1099.

[11] Itah, Zeynep, et al. "HER2-driven breast cancer suppression by the JNK signaling pathway." *Proceedings of the National Academy of Sciences* 120.4 (2023): e2218373120.

[12] Adelson, Kerin, et al. "Randomized phase II trial of fulvestrant alone or in combination with bortezomib in hormone receptor-positive metastatic breast cancer resistant to aromatase inhibitors: a New York Cancer Consortium trial." *NPJ Breast Cancer* 2.1 (2016): 1-6.

[13] Yi, Yong Weon, et al. "Dual inhibition of EGFR and MET induces synthetic lethality in triple-negative breast cancer cells through downregulation of ribosomal protein S6." *International Journal of Oncology* 47.1 (2015): 122-132.
